# Antibacterial Compounds of Canadian Honeys Target Bacterial Cell Wall Inducing Phenotype Changes, Growth Inhibition and Cell Lysis That Resemble Action of β-Lactam Antibiotics

**DOI:** 10.1371/journal.pone.0106967

**Published:** 2014-09-05

**Authors:** Katrina Brudzynski, Calvin Sjaarda

**Affiliations:** Bee-Biomedicals Inc., Drug Discovery and Development Department, St. Catharines, Ontario, Canada; Rockefeller University, United States of America

## Abstract

Honeys show a desirable broad spectrum activity against Gram-positive and negative bacteria making antibacterial activity an intrinsic property of honey and a desirable source for new drug development. The cellular targets and underlying mechanism of action of honey antibacterial compounds remain largely unknown. To facilitate the target discovery, we employed a method of phenotypic profiling by directly comparing morphological changes in *Escherichia coli* induced by honeys to that of ampicillin, the cell wall-active β-lactam of known mechanism of action. Firstly, we demonstrated the purity of tested honeys from potential β-lactam contaminations using quantitative LC-ESI-MS. Exposure of log-phase *E. coli* to honey or ampicillin resulted in time- and concentration-dependent changes in bacterial cell shape with the appearance of filamentous phenotypes at sub-inhibitory concentrations and spheroplasts at the MBC. Cell wall destruction by both agents, clearly visible on microscopic micrographs, was accompanied by increased permeability of the lipopolysaccharide outer membrane as indicated by fluorescence-activated cell sorting (FACS). More than 90% *E. coli* exposed to honey or ampicillin became permeable to propidium iodide. Consistently with the FACS results, both honey-treated and ampicillin-treated *E. coli* cells released lipopolysaccharide endotoxins at comparable levels, which were significantly higher than controls (p<0.0001). *E. coli* cells transformed with the ampicillin-resistance gene (β–lactamase) remained sensitive to honey, displayed the same level of cytotoxicity, cell shape changes and endotoxin release as ampicillin-sensitive cells. As expected, β–lactamase protected the host cell from antibacterial action of ampicillin. Thus, both honey and ampicillin induced similar structural changes to the cell wall and LPS and that this ability underlies antibacterial activities of both agents. Since the cell wall is critical for cell growth and survival, honey active compounds would be highly applicable for therapeutic purposes while differences in the mode of action between honey and ampicillin may provide clinical advantage in eradicating β-lactam-resistant pathogens.

## Introduction

Research into antibacterial properties of honey and compounds involved in this activity provided a significant opportunity to discover potential novel lead compounds for the development of antibacterial therapy. Both raw and sterilized (pasteurized or irradiated) honeys showed a desirable broad spectrum activity against Gram-positive and Gram-negative bacteria [1], including medically important pathogens such as *Burkholderia cepacia* [2], *Pseudomonas aeruginosa* [3], [4], *Salmonella enterica, Serratia marcescens, Escherichia coli, Klebsiella pneumoniae* [5], *Staphylococcus aureus*, *Salmonella typhymurium*, *Shigella sonnei* [6] and *Streptococcus pyogenes* [7]. Moreover, several multidrug-resistant bacteria, including methicillin-resistant *Staphylococcus aureus* (MRSA) and vancomycin-resistant *Enterococci* (VRE) showed susceptibility to honey action [8], [9]. Studies on activity-associated biomarkers revealed that hydrogen peroxide and methylglyoxal significantly contribute to honey antibacterial activity [10]–[13]. However, neither action of hydrogen peroxide nor methylglyoxal could account for the total antibacterial activity of honey, since their removal did not completely abrogate honey cytotoxicity [6], [11], [14].

It is undeniable fact that almost all honeys exert at least bacteriostatic activities independently of their botanical or geographical origins making antibacterial activity an intrinsic property of honey and a desirable source for new drug development. We hypothesized that these putative, antibacterial molecule(s) must have been able to recognize and damage cellular targets that are crucial for bacterial viability. Therefore, we aimed at discovery of targets for these compounds and underlying mechanism of action in order to facilitate progress in elucidating the basis for honey antibacterial activity. In our target- based approach, we employed a method of phenotypic profiling [15] by directly comparing morphological changes in *Escherichia coli* evoked by honeys to those evoked by antibiotics of known mechanism of action. We have chosen in this study ampicillin, a cell wall-active β-lactam, as a drug model.

The cellular target of β-lactam is the peptidoglycan (PG) that surrounds on the outside the plasma membrane of bacterium and is linked to the lipopolysaccharide layer of outer membrane in Gram-negative bacteria. PG is the mesh-like polymer of β-(1, 4) linked *N*-acetylglucosamine and *N*-acetylmuramic acid crosslinked by short three to five amino acid stem peptides. PG synthesis requires penicillin-binding proteins (PBPs) that functions as DD-transpeptidases (DD-TPases) to crosslink the peptides to glycan chains, polymerized by glycotransferases (GTases) [16], [17]. A concerted action of several *E. coli* PBPs is required for a formation of PG sacculus and the characteristic rod shape. In general, the activities of PBP1A, PBP1B have been shown to be associated with the cell elongation while PBP2 and PBP3 are responsible for maintaining the rod-shape structure and a septation during cell division, respectively [16]–[19]. β-Lactams covalently bind PBPs (DD-TPases) thereby inhibiting transpeptidation, the final stage of peptidoglycan synthesis. β-Lactams binding to PBPs result in a series of well-defined, characteristic morphological changes: inactivation of PBP2 lead to formation of spherical cells, inhibition of PBP3 results in formation of long filaments while inactivation of PBP 1A and 1B results in rapid cell lysis [16]–[19], [20], [21].We hypothesized that these well characterized morphological changes caused by β-lactams could serve as a reference point to compare with changes evoked by honey components. This phenotypic profiling in conjunctions with comparative analyses of the growth rate and cell viability of different phenotypes could provide an important clue as to the cellular targets recognized by honey antibacterial molecule(s). The target identification is an important starting point allowing and guiding our future discovery of active compounds and their mode of action.

## Results

### 1. A comparison of *E. coli* susceptibility to honey and ampicillin

The *E. coli* growth inhibition and time-kill kinetics evoked by exposure to honeys and ampicillin were analyzed using broth microdilution assay and a standard plate count. The MIC_90_, MBC_100_ values are summarized in Table 1. Each reported value is based on twelve to fifteen replicates from three independent experiments.

**Table 1:** Susceptibility of *E. coli* to honeys.

	Buckwheat Honey H208	Wildflower Honey H210	Ampicillin
MIC	6.25% (w/v)	6.25% (w/v)	0.625 µg/ml
MBC	6.25% (w/v)	12.5% (w/v)	0.625 µg/ml

### 2. A comparison of functional dynamics of ampicillin and honey on *E. coli*

**Figure 1:**
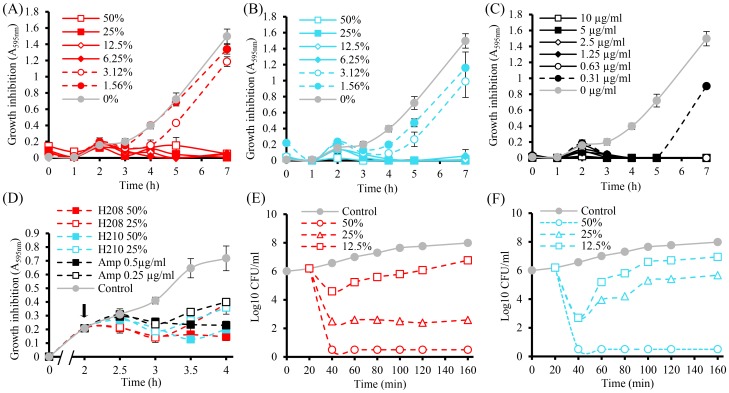
Kinetics of growth inhibition of lag-phase and log-phase *E. coli* exposed to honeys and ampicillin (A,B,C) and (D), respectively, and time-kill kinetics (E, F). The arrow indicates the application time of honeys or ampicillin into log-phase *E. coli*. In A to D, each point represents the mean ± standard deviation (n = 9), in E and F (n = 4).

Growth inhibition and bactericidal action of β-lactams depends on the bacterial growth phase and correlates with generation time [22]. To compare the growth dynamics and viability of *E. coli* exposed to honey, the lag-phase cultures were either directly challenged with honey and ampicillin (Fig. 1A, B, and C) or were first incubated to reach the exponential phase (0.2 A_595_nm) before adding these two agents (Fig. 1D). In both cases, the cultures were treated with honeys at concentrations ranging from 50% to 3.125% (w/v) and ampicillin (1.25 µg/ml to 0.31 µg/ml).

Starting from the lag-phase, growth curves of *E. coli* (10^6^ CFU/ml) showed a latent period of about 2 h during which an increase in absorbance was observed in all tested samples; in untreated, honey-treated and ampicillin-treated cultures (Fig.1A, B and C). The reduction of growth by honey and ampicillin occurred immediately *after* the cells entered the early exponential phase and started to divide, suggesting that honey targeted events related to initiation of growth and cell division.

Honeys at and above their 1xMICs (6.25% v/v) were inhibitory for the entire 18 h of incubation (Fig.1A and B). Similarly, ampicillin at concentrations >0.625 µg/ml completely blocked *E. coli* growth (Fig.1C). No viable cells were observed in the standard plate count when samples from incubation wells were plated onto agar plates. In contrast, sub-inhibitory honey concentrations (3.12% (w/v) and 1.5% (w/v)), were unable to kill the cells but were capable to reduce the growth (Fig.1A, B, C, lag-phase and D, log-phase *E. coli*).

Application of honeys into the log-phase *E. coli* cultures caused the reduction of growth in all treated samples in the first 20 to 30 min that correspond to one generation time (Fig. 1D). Thereafter, the increase in absorbance indicated the gradual loss of honeys' inhibitory efficiencies. Honeys at concentrations 12.5% (w/v) (H208) and 25%w/v (H210) caused a reduction of viable cells by 2 to 3log_10_, respectively, within 1 to 2 h incubation time (Fig. 1D). The >5log_10_ reduction of bacterial counts required 2 to 4x lag-phase MICs, corresponding to log-phase MICs 25% and 50%w/v for honey H208 and H210, respectively (Fig. 1E and F).

These results suggested that honey effects on *E. coli* were dependent on the MIC/MBC, bacterial growth phase and incubation time (the duration of exposure of *E. coli* to honey). The picture that emerges from these results indicates that the initial rapid decline in bacterial counts in both lag-phase and log-phase cultures resulted from the successful binding of honey antibacterial components to its target. Loss of bactericidal potency occurred when the concentration of unbound antibacterial component fell below the MBC level (25% (w/v) and 50% (w/v) for H208 and H210, respectively, for log-phase cultures). Although the bactericidal activity was lost, honeys continued to be inhibitory (Fig. 1D). Thus, honey antibacterial effects were concentration-dependent; when the effective concentrations of honey antibacterial compounds decreased below the MBC/MIC levels, the cell viability and growth were not longer affected and *E. coli* proliferation resumed.

On the other hand, exposure of log-phase *E. coli* to ampicillin in concentrations ranging from 10 µg/ml to 0.31 µg/ml caused a complete growth inhibition at the MIC/MBC 0.625 µg/ml (Fig. 1C and D) and bacterial death. No viable cells were observed in a standard plate count.

The results indicated that both honey and ampicillin recognized and interfered with the cellular targets of *E. coli* cell that were important for sustained growth and multiplications. The observed differences in rates of bacterial killing in the lag versus log-phase cultures and differences in bactericidal potency between honey and ampicillin suggested that the bactericidal effects of both agents might results from independent killing modes.

### 3. A comparison of morphological dynamics in *E. coli* induced by honey and ampicillin

**Figure 2:**
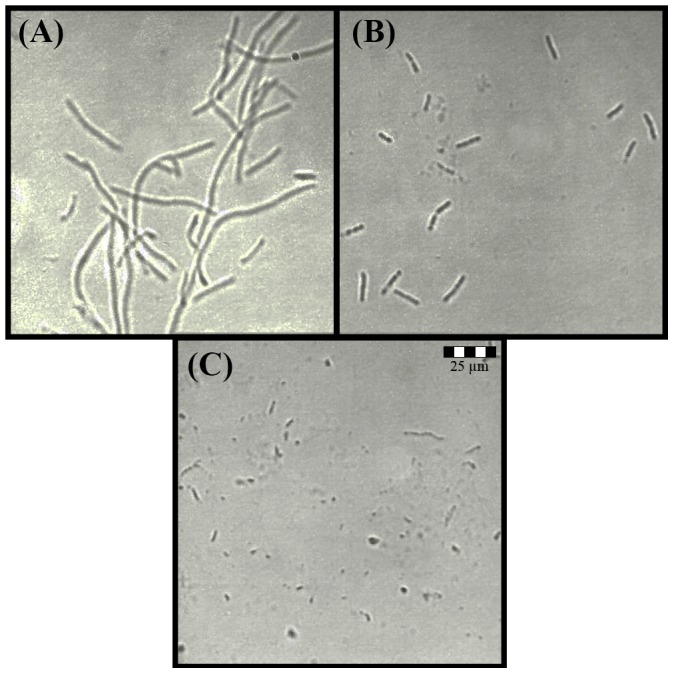
Concentration-dependent changes in *E. coli* phenotype after treatment with ampicillin. Ampicillin concentrations: (A) 0. 31 µg/ml, (B) 0.625 µg/ml and (C) 1.25 µg/ml. The size-scale is the same for all micrographs in the figure, obtained under the same 400x magnification.

The ampicillin mode of action involves inhibition of PG synthesis that, in turn, changes the structural integrity of the cell wall resulting in characteristic morphological alterations. We confirmed ampicillin effects on *E. coli* morphology at concentrations used in our growth inhibition and time-kill kinetics. The addition of 0.3-2.5 µg/ml ampicillin to the log-phase *E. coli* cultures resulted in a dose-dependent phenotype changes (Fig.2). Increased filamentation was observed at ampicillin concentration of 0.31 µg/ml, the filament lysis and formation of spheroplasts was visible at 0.62 µg/ml, while spheroplast phenotypes were predominate forms at concentration of 1.25 µg/ml to 2.5 µg/ml (Fig. 2).

**Figure 3:**
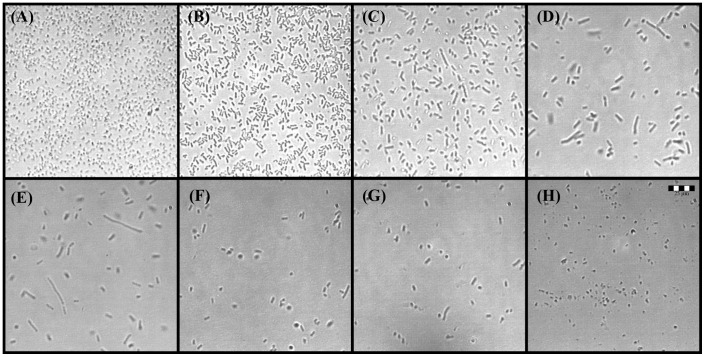
Time-dependent phenotypic changes observed after application of honey to log phase *E. coli* cultures. The changes paralleled the growth kinetics shown in Fig. 1D. (A) lag phase *E. coli* culture, (B) log phase *E. coli* culture at the time of honey application, (C) *E. coli* phenotypes after 30 min incubation with honey H208 (12.5% v/v), (D). *E. coli* phenotypes observed after 1 h incubation with honey H210 (12.5% v/v), (F) and (G) *E. coli* spheroplasts formed after 2 h incubation with honey H208 and H210 at 50% v/v, (H) spheroplasts observed after 18 h incubation, lag phase. The size-scale is the same for all micrographs in the figure, obtained under the same 400x magnification.

To investigate whether honey could alter cell morphology in a similar manner, samples taken at regular time intervals from incubation wells, during incubation of honeys with log-phase *E. coli,* were simultaneously analyzed for their morphology, growth inhibition and time-kill kinetics. Firstly, the initiation of growth of *E. coli* cells from the lag phase was seen as the transition from coccoidal forms of the stationary phase to the typical 2–4 µm short rods of normal, growing *E. coli* (Fig.3A and B). Addition of honey during initiation of growth (after 2h incubation) correlated with the formation of a heterogeneous population of phenotypes consisting of rods, filaments and spheroidal cells (Fig. 3C). The decreased number of cells observed on microscopic micrographs during the first hour after honey application to the culture, was consistent with a rapid decline of cell viability observed in time-kill kinetics (Fig. 3D and E and Fig. 1E and F). From the initial heterogeneous cell population, the cells that survived consisted of longer rods and filaments, indicative of the inhibition of septation and cell division (Fig. 3D and E). After 18 h incubation, spheroplasts of different sizes, mini cells and cell debris were the main components observed in honey-treated samples (Fig.3F-H). The appearance of these phenotypes correlated with the >5log_10_ reduction of bacterial counts by honey H208 at the MBC as shown in Fig. 1 E and F.

**Figure 4:**
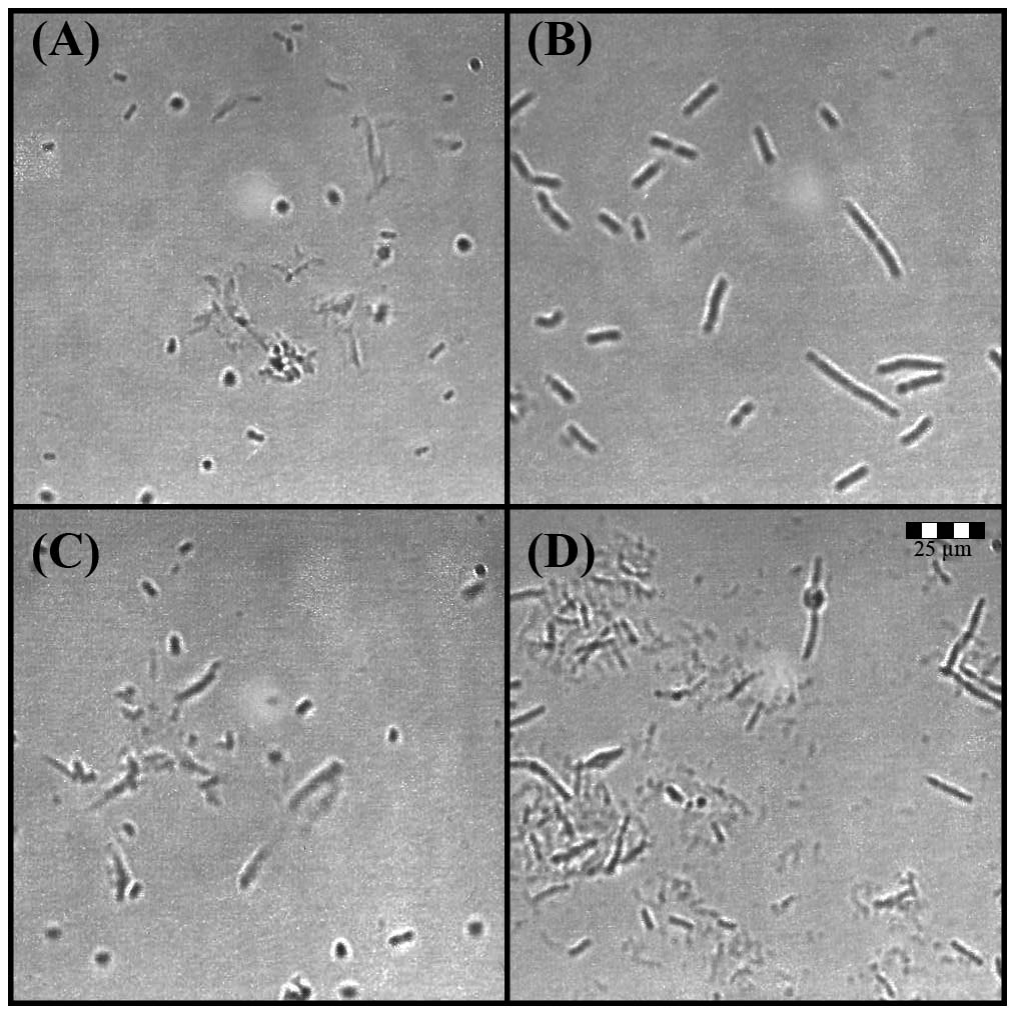
Concentration-dependent *E. coli* cell lysis induced by honey (A and C) and ampicillin (B and D) at sub-inhibitory (A and B) and inhibitory concentrations (C and D). The size-scale is the same for all micrographs in the figure, obtained under the same 400x magnification.

**Figure 5:**
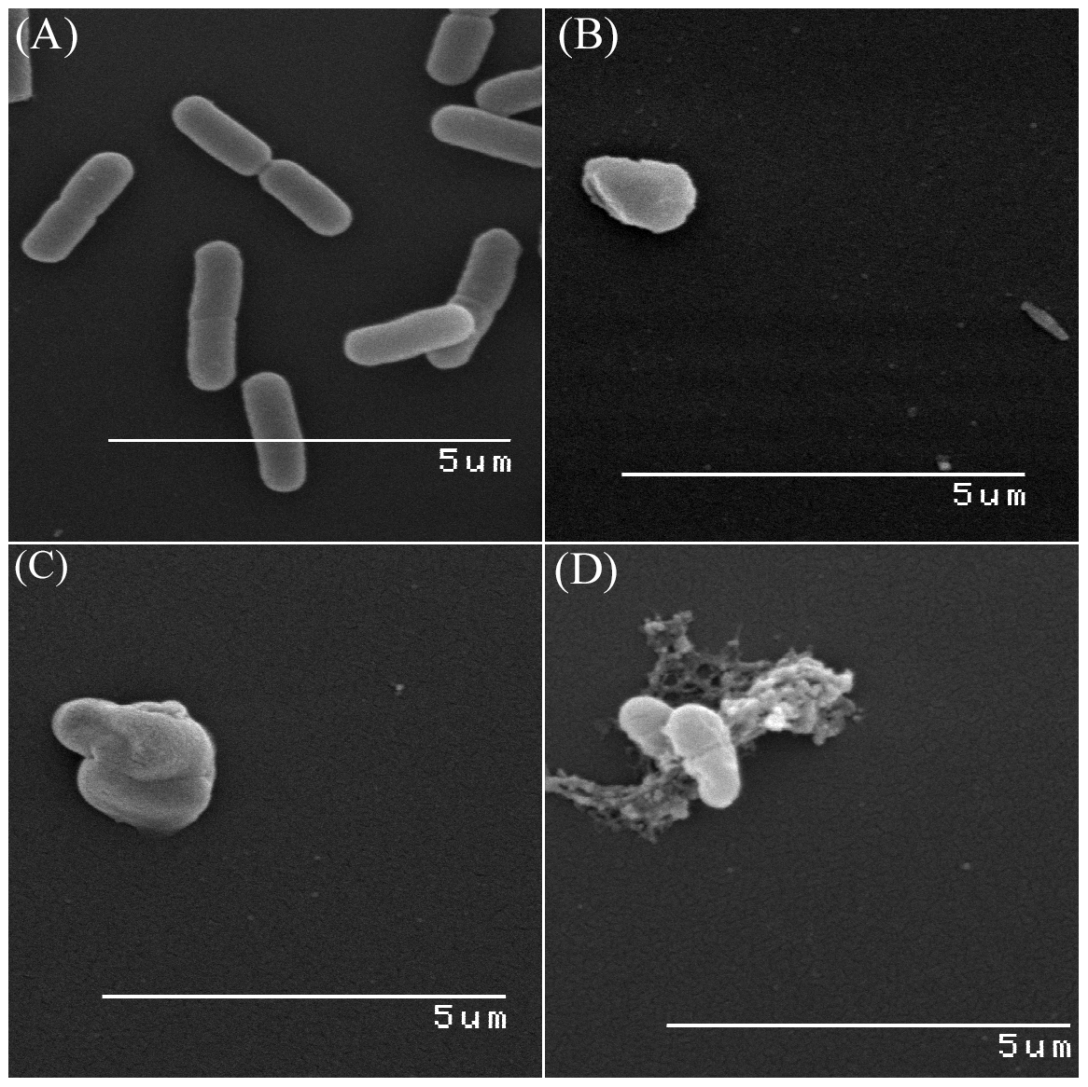
SEM of *E.coli* spheroplasts generated by ampicillin and honey-treatments. Log-phase *E. coli* cells were treated with bactericidal concentrations of ampicillin (2.5 µg/ml) and honey H208 (25% w/v) for 3 hr at 37°C. Cells were harvested and glutaraldehyde-fixed (see Materials and Methods). (A). *E. coli* control, (B). *E. coli* cels after treatment with ampicillin and (C). *E. coli* cell after treatment with honey and (D) honey-induced spheroplast lysis.

These results indicated that, similarly to ampcillin, honey action affected the cell wall structure that is responsible for *E. coli* cell shape. As in the case of ampicillin, the changes were dose-dependent and related to the appearance of the filamentous phenotypes at sub-inhibitory concentrations and spheroplast formation at the MBC (Fig.4). Morphological similarities between *E. coli* spheroplasts formed by ampicillin and honey-treatment were also supported by the SEM images (Fig.5). Importantly, spheroplasts generated by honey-treatment were prone to lysis (Fig. 5).

The altered *E. coli* morphology critically affected *E. coli* growth and cell viability as indicated in the functional assays (Fig. 1). Thus, it became clearly revealed that honey compounds targeted the cell wall of *E. coli* causing structural changes and that the cell wall damaging effect constituted the mechanism underlying the antibacterial effects of honey and ampicillin.

### 4. A comparison of effects of honey and ampicillin on a permeability of *E. coli* outer membrane

Lipopolysaccharide (LPS) of outer membrane in Gram-negative bacteria is a permeability barrier. LPS is cross-bridged to the peptidoglycan of the cell wall [23] and the loss of the cell wall integrity induced by ampicillin affects also the LPS integrity [23], [24]. To compare the changes in LPS permeability induced by honey and ampicillin in a quantitative way, we used forward and side scatter measurements of fluorescence-activated cell sorting using two nucleic acid-specific stains; membrane-permeable Syto9 and membrane-impermeable propidium iodide (PI).

**Figure 6:**
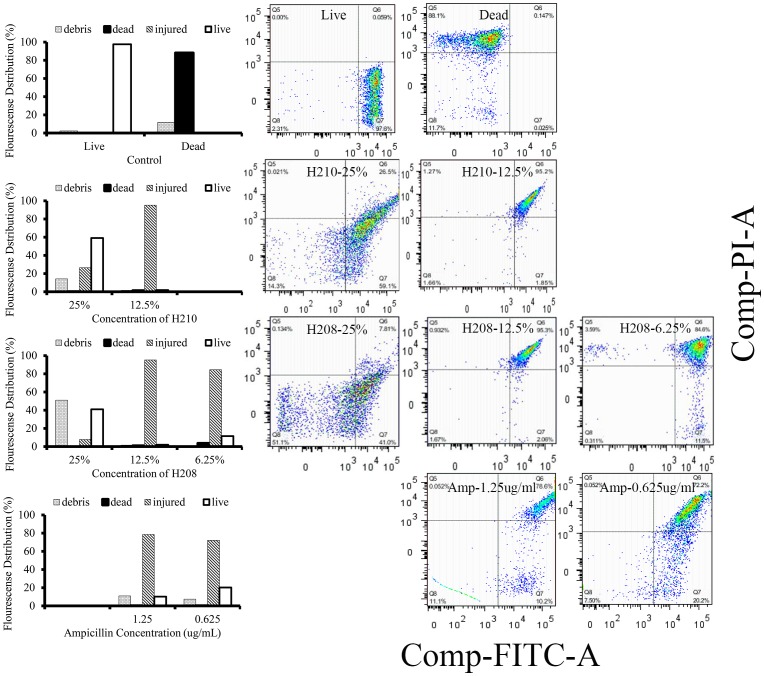
Effects of ampicillin and honey treatments of log-phase *E. coli* on lipopolysaccharide membrane permeability using fluorescence-activated flow cytometry. The distribution of *E. coli* populations in each of the four quadrants was quantified by the forward scatter of green fluorescence of Syto9 (as FITC-A) versus side scatter of red fluorescence of PI. The left panel shows the percentage of dead (PI positive), live (Syto9-positive) and PI/Syto9-double positive, injured cells after *E. coli* treatment with honeys H210, H208 and ampicillin.

After 3 h exposure of log-phase *E. coli* cultures to honey or ampicillin, the stained cells were sorted into four distinct *E. coli* subpopulations: PI- positive (dead cells), Syto-positive (live cells), Syto9/PI double positive cells as injured, cells and Syto9/PI double negative cells that may include cell debris. The FACS results showed that the distribution of fluorescence was striking comparable between *E. coli* exposed to honey or ampicillin (Fig.6). At sub-inhibitory concentrations, both agents caused a marked increase in double-stained PI/Syto9 cells. The percent of these injured cells ranged from 95.5% to 78.6% for honeys (at concentrations of 25% and 12.5% w/w) and ampicillin (at concentration 1.25 ug/ml), respectively (Fig. 6). Thus, both ampicillin and honey actions inflicted severe damages to the *E. coli* LPS with comparable efficacies.

Unexpectedly, exposure of log phase *E. coli* to bactericidal concentrations of honeys produced 59% to 41% of Syto9–positive (live cells), respectively, and only limited amounts of injured cells (26% to 7. 8%), respectively, while no PI-positive (dead) cells were visible (Fig. 6). Despite the presence of over 50% of Syto9-positive, live cells, these cell populations did not grow well when plated on the agar plates: honey H210 at concentration of 25% w/v showed 2log_10_ CFU/ml reduction of viable cells, while honey H208 at the concentration of 25% w/v reduced viable cells by >4log_10_ CFU/ml with no colony observed at the concentration of 50%w/v of both honeys (Fig. 1E). Under microscope, they presented themselves as spheroidal forms and mini cells (Fig. 4H). The spheroidal forms devoid of peptidoglycan layer (spheroplasts) and mini cell populations are often suggested to represent the antibiotic-tolerant state of viable but nonculturable cells (VBNC) [25]–[28] and persisters cells [29], respectively. These morphological forms are known, protective phenotypes of *E. coli* observed under stressful conditions [25], [26], [29]. Whether the spheroidal forms represent VBNC requires further studies to clarify the apparent discrepancy between the plate count and flow cytometry results.

The results of FACS showed that sub-inhibitory concentrations of honey and ampicillin drastically increased the LPS permeability allowing a penetration of PI into the cell. The LPS disintegration caused by honey action was yet another, previously unknown phenomenon.

### 5. A comparison of endotoxin release from *E. coli* induced by honey and ampicillin

**Figure 7:**
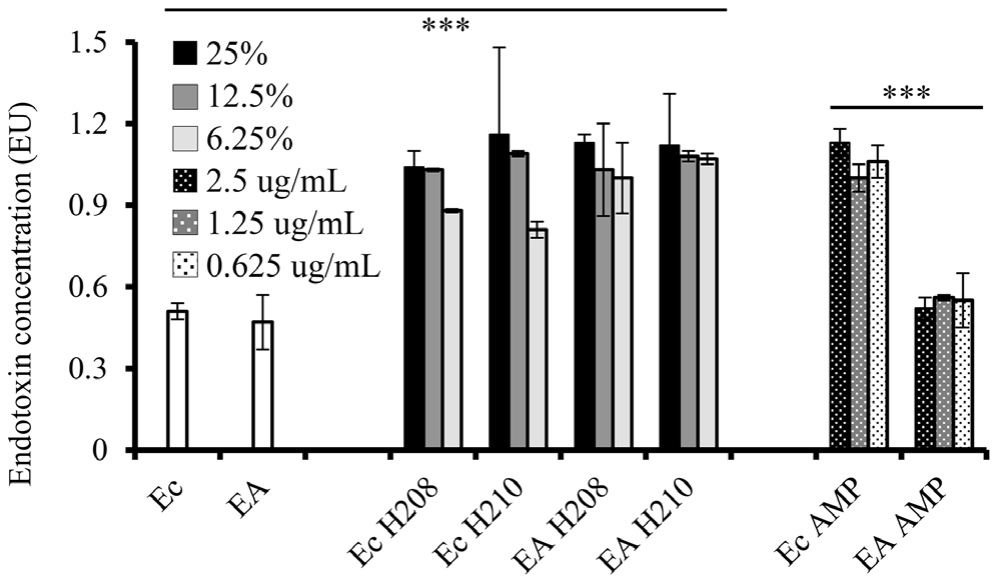
Endotoxin release from ampicillin-sensitive and ampicillin-resistant *E. coli* cells after exposure to different concentrations of honey and ampicillin. (EA: ampicillin-resistant *E. col*, Ec-*E. coli*). The bars represent the mean ± standard deviation (n = 4). See text for statistical results. *** - p<0.0001.

The LPS disintegration in *E. coli* induced by ampicillin is accompanied by a simultaneous release of high levels of endotoxins [23], [24]. We asked whether LPS release would also be observed following *E. coli* exposure to honey. The log-phase cells were incubated for 3 h with honeys H210 and H208 at concentrations ranging from 25% to 6.25% w/v, respectively and with ampicillin at concentrations 0.625 µg/ml to 2.5 µg/ml. Free endotoxin levels in cell supernatants were quantified using a chromogenic Limulus amebocyte lysate (LAL assay) using a constructed standard curve with linearity of R^2^ = 0.9912. The endotoxin levels released from *E. coli* were significantly higher in honey and ampicillin-treated groups than in untreated *E. coli* controls (p<0.0001) (Fig.7).

**Figure 8:**
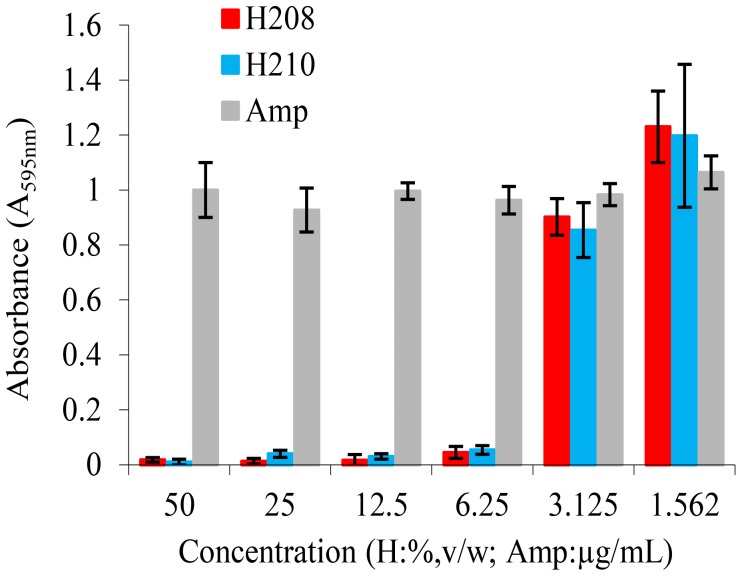
Susceptibility of ampicillin-resistant *E. coli* to honey and ampicillin. The bars represent the mean ± standard deviation (n = 9).

**Figure 9:**
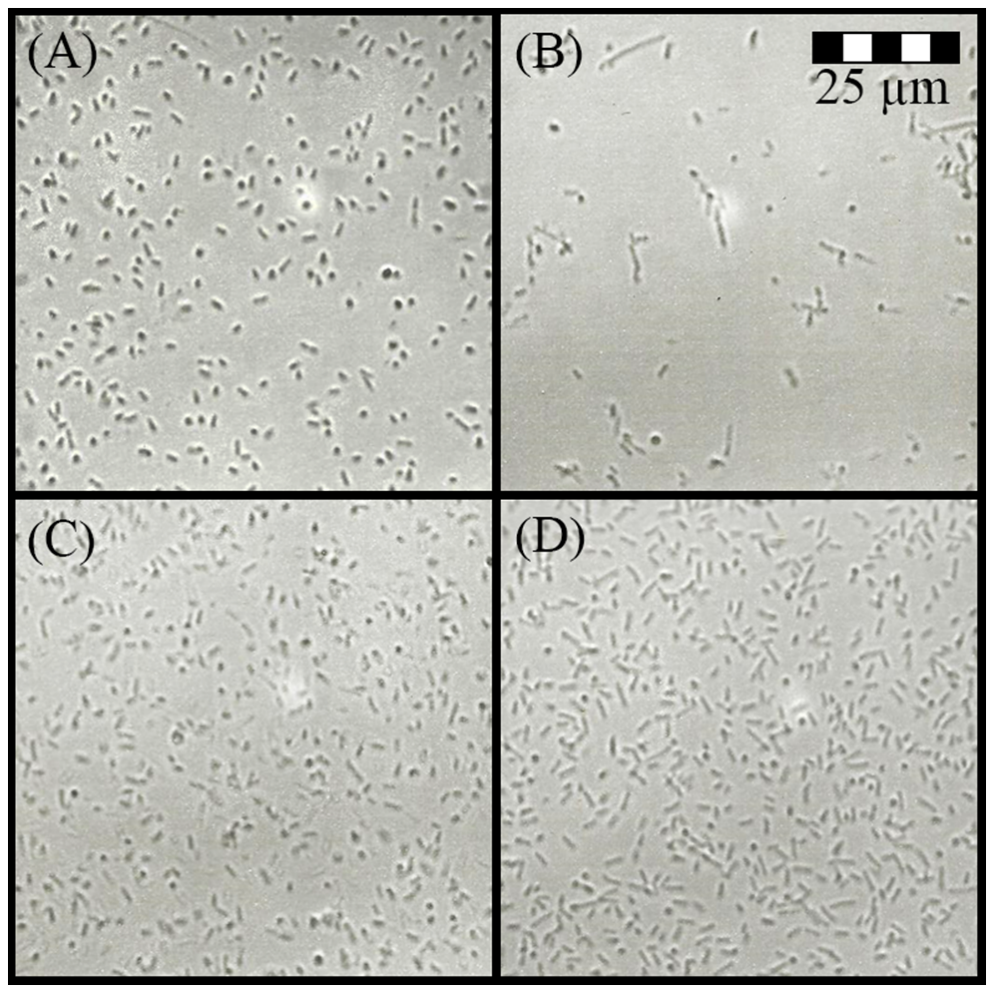
Morphological changes in ampicillin-resistant *E. coli* after exposure to honey or ampicillin. Ampicillin-resistant *E. coli* before (A) and after treatment with honey, H210, 25%w/v, (B) and ampicillin-resistant *E. coli* before (C) and after treatment with 2.5 ug/ml of ampicillin (D). The size-scale is the same for all micrographs in the figure, obtained under the same 400x magnification.

To generate an additional, negative control for endotoxin release, we transformed *E. coli* with plasmid-encoded ampicillin-resistance gene coding for β-lactamase, to create a model of β-lactam resistance. Firstly, we evaluated the susceptibility of *amp*-resistant *E. coli* to honey antibacterial compounds. As expected, the growth of *amp*-resistant *E. coli* (EA) was not affected by the presence of ampicillin in the culture media (Fig.8). The insensitivity of EA to ampicillin was also reflected by the lack of phenotype changes in the presence of this antibiotic (Fig. 9). In contrast, honeys inhibited the growth of EA with the same efficacy as ampicillin-sensitive cells (Fig.8). The honeys MIC_90_ 6.25%w/v against lag-phase EA was not different from those of ampicillin-sensitive *E. coli* (Fig.8 and Table 1). The cell wall lysis induced by honey was clearly evident in both *amp*-resistant and sensitive *E. coli* (Fig.9). It became apparent that the destruction of bacterial cell wall by honey and ampicilln occurred by independent modes of action; the presence of β-lactamase gene did not change *E. coli* susceptibility to honey.

Next, we investigated the effects of ampicillin resistance on the level of endotoxin release. When *amp*-resistant (EA) and *amp*–sensitive *E. coli* (Ec) were exposed to honeys, the levels of endotoxin liberated were essentially identical (Fig. 7, bars Ec H208, Ec H210, EA H208, and EA H210, differences n.s.). However, the amount of endotoxin release from honey-treated groups was significantly higher from that of untreated controls (honey-treated groups versus controls Ec and EA), t[50] = 8.99, p<0.0001). Moreover, amicillin-resistance had no effects on honey-induced endotoxin release. In contrast, ampicillin treatment of *amp*-resistant *E. coli* did not increase endotoxin release over the levels observed in control cells (Fig. 7, control groups Ec and EA versus EA AMP did not significantly differ (t[9] = 2.1, n.s.) and no morphological changes were observed (Fig. 9). At the same time, ampicillin caused significantly higher amounts of endotoxin release from ampicillin-sensitive than ampicillin-resistant cells (Fig. 6, the bars Ec AMP vs. bars EA AMP, compared jointly, t-test, t [22] = 21.9, p<0.0001) (Fig. 7).

Thus, endotoxin release from the LPS of ampicillin–sensitive *E. coli* by honey was similar to that of ampicillin but the modes of actions were different. The ampicillin-resistance gene provided “protection” against ampicillin action on the cell wall and LPS. The differences in susceptibility patterns against *amp*-resistant *E. coli* between honey and ampicillin may be of clinical significance in the treatment of β-lactam-resistant *E. coli*.

### 6. Quantitative analysis of potential β-lactam contaminations in tested honeys

**Figure 10:**
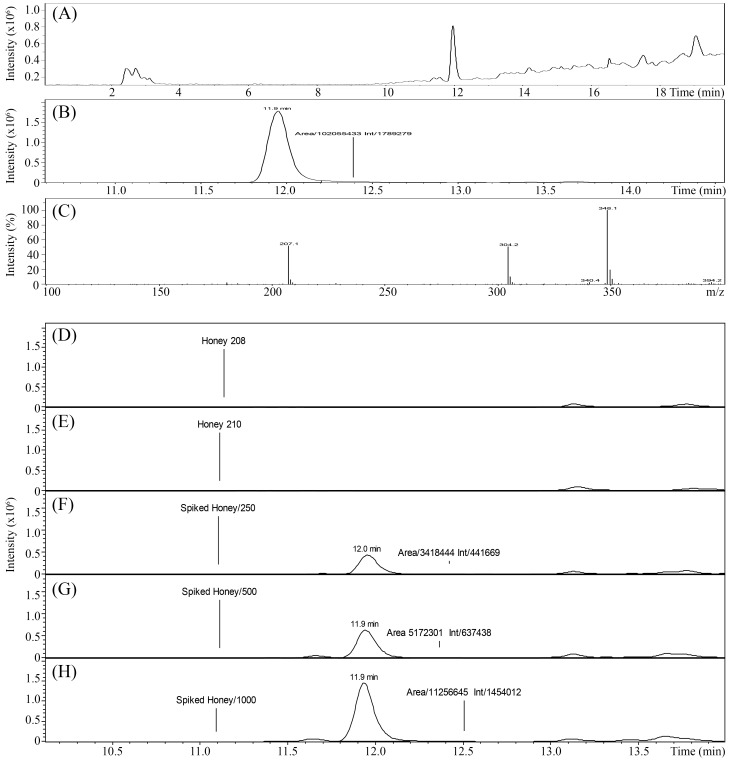
Total Ion Current (TIC) and MS full-scan analysis of ampicillin standard obtained from LC/ESI/MS in negative mode. The *m/z* 348.1 eluting at RT 11.9 min was used for quantitation of antibiotic traces in honeys H208 and H210. Honey H208 spiked with amplicillin at 250 ng, ml, 500 ng/ml and 1000 ng/ml served as internal standards.

To analyze a potential contamination of honey with β-lactam antibiotics, honey samples were extracted with ethyl acetate, purified using SPE procedures and analyzed using LC-ESI-MS. The total ion chromatogram and mass spectrum of ampicillin standard (C16H19N3O4S, molecular weight 349.41) gave a main mass ion [M-H]^-^ m/z 348.1 at retention time (RT) 11.9 min (Fig. 10). This mass ion was used to screen honeys for antibiotic traces. Honeys spiked with amplicillin at 250 ng/ml, 500 ng/ml and 1000 ng/ml served as internal standards and produced the mean peak area linearity of R^2^ = 0.907. The LC-ESI-MS method was sensitive to detect nanogram quantities of ampicillin in spiked honeys, but no traces of antibiotic were found in non-spiked honeys H208 and H210 (Fig. 10). Thus, the results showed that the membrane damaging and bacteriolytic effects of H208 and H210 did not result from the potential presence of β-lactam traces.

## Discussion

The method of phenotypic profiling combined with functional and morphological analyses of *E. coli* exposed to honey and ampicillin allowed uncovering previously unknown targets for honey compounds and allowed understanding the antibacterial effects they produced. Targeting of the cell wall and the lipopolysaccharide outer membrane by honey compounds is arguably the most important finding of this study. The cell wall peptidoglycan is vital for maintaining cell shape and for sustaining bacterial growth and reproduction [23], [30], [31], [37], [38]. We showed here that honey, similarly to ampicillin, caused cell shape changes that resulted from the loss of cell wall integrity. Using SEM and an image-based microscopy in combination with functional assays we have shown that exposure of *E. coli* to honey disrupted the cell wall and caused morphological changes that included filamentation, filament lysis, formation of spheroplasts and ultimately, cell lysis. The suite of phenotypic changes matched a specific action of ampicillin on the cell wall. Moreover, the cell shape changes occurred in time- and concentrations-dependent manner and correlated with the honey MIC and MBCs. The new phenotypes became visible immediately upon initiation of *E. coli* growth in the presence of honey and were followed by growth cessation and a rapid decrease in viable cell counts. Thus, targeting the cell wall and its damage by honey compounds was accountable for honey antibacterial effects. These findings advance our understanding of the reasons behind honey antibacterial activity.

The mechanism of antibacterial activity of ampicillin is linked to an inhibition of cell wall synthesis by a direct binding to penicillin-binding protein, PBP1A, and inactivating its transpeptidase activity [16], [20], [21]. High concentrations of ampicillin and other β-lactam antibiotics usually inactivate transpeptidase activity in all PBPs. This generates spheroplast phenotype with up-regulated expression of genes involved in spheroplast lysis [35], [36]. Rapid autolysis ensues. At sub-inhibitory concentrations however, activity of PBP2 responsible for cell elongation might be preserved and produce filamentous phenotype [20], [32]–[34]. Similarly, we have shown here that honey-induced formation of spheroidal forms occurred at the MBC during the first 20 to 30 min after honey application to log-phase *E. co*li culture, that is, during one generation time. The drastic reduction of cell viability (up to 5log_10_ CFU/ml) indicated that injury done to *E. coli* cells was critical and beyond recovery. Scanning electron microscopy clearly showed spheroplast lysis. Despite the overlapping phenotypic changes and functional outcomes induced by honey and ampicillin, the mode of honey action on cell wall remains unknown. Whether cell shape changes caused by honey were the results of inhibition of the cell wall synthesis or the results of a direct physical injury to the cell wall integrity have to be further investigated.

The exposure of *E. coli* to honey and ampicillin also affected structural integrity of lipopolysaccharide (LPS) of outer membrane that regulates the cell permeability. The outer membrane is impermeable to large molecules and hydrophobic compounds. However, fluorescence-activated cell sorting (FACS) showed that over 90% of honey-treated *E. coli* became permeable to propidium iodide suggesting that the LPS barrier was compromised. Although the increase in membrane permeability to nucleic acid dyes of bacterial cell exposed to β-lactam antibiotics has been previously described [40], [41], the LPS destruction by honey is a new, previously unknown phenomenon.

Consistent with FACS results, honey-treated cells released LPS endotoxins at the levels comparable to that of ampicillin-treated cells but significantly higher than that of untreated *E. coli* as measured using Limulus Amoebocyte Lysate (LAL) assay. Endotoxins are LPS constituents of the outer membrane of cell wall of gram-negative bacteria and their toxicity is associated with the Lipid A which cross-bridge the outer membrane with peptidoglycan layer [23]. Cell lysis caused by exposure of Gram-negative bacteria to β-lactam antibiotics increases release of endotoxins [42]. We have shown here that honey-induced cell wall damage was also associated with endotoxin release. Since cell lysis requires both cell wall and LPS disintegration, this provided additional strong support that the cell wall and lipopolysaccharide of outer membrane were targets for honey action. Disruption of these targets was responsible for the antibacterial effects of honey.

Although honey and ampicillin targeted *E. coli*'s cell wall and induced overlapping changes in phenotype, suppression of cell division and reduction of cell viability, the modes of their actions were likely different. In our ampicillin-resistance model of *E. coli* transformed with plasmid containing ampicillin-resistance gene, β-lactamase production prevented cytotoxic effects of ampicillin and endotoxin release but did not affect *E. coli* sensitivity to honey antibacterial effects.

To the best of our knowledge, there are no reports that identify bacterial cell wall as a specific cellular target for honey compounds. The cell wall and LPS are “new targets” that have been suspected but not yet definitively established. The cell lysis, specifically of Gram-negative bacteria, has been recently observed in studies aiming at elucidation of molecule(s) responsible for bactericidal effects in honey of *Leptospermum spp* (manuka honey) [14], [39]. In *Pseudomonas aeruginosa,* manuka honey induced phenotype changes, growth inhibition and cell lysis [39], [43]. On genetic level, it affected cell survival by reducing the expression of three microcolony-forming genes [43]. Specifically, the reduced expression of *oprF* gene, seen after 60 min incubation of *P. aeruginosa* with manuka honey, suggested that the gene was a target for this honey. It has been further postulated that the possible reduction of protein product of the *oprF* gene, localized to outer membrane, might cause a significant stress and facilitates its disruption [43]. In contrast to manuka honey, we have shown in this study that buckwheat and wildflower honeys induced a rapid, concentration and time-dependent disruption of the cell wall, reducing bacterial counts by >5log10 CFU/ml within the first 30 min of honey application to bacterial cultures. The differences in killing kinetics and efficiency may suggest that manuka and Canadian honeys did not target the same structures, and that these targets might be located at different cellular compartments.

From this study, Canadian honeys have emerged as an antibacterial agent active against the bacterial cell wall. Since the cell wall plays a fundamental role in the cell growth and survival, the compounds responsible for the cell wall damaging effects would be highly applicable for therapeutic purposes. Moreover, differences in their mode of action from ampicillin may be advantageous in eradicating β-lactam-resistant strains.

## Materials and Methods

### Honeys

Honeys were donated by Canadian beekeepers and consisted of mixed buckwheat honey (H208) (fagopyrum/solidago/brassica) and wildflower honey (H210) (rhamnaceae/ligustrum/trifolium) as indicated by melissopalynology. The honeys were collected during 2012/13 season.

### Bacterial strains and growth cultures

Standard strains of *Escherichia coli* (ATCC 14948) purchased from Thermo Fisher Scientific Remel Products (Lenexa, KS 66215) were grown in Mueller-Hinton Broth (MHB) (Difco Laboratories) overnight in a shaking water bath at 37°C. Overnight cultures were diluted with broth to the equivalent of the 0.5 McFarland standard.

### Broth microdilution assay and determination of the MIC

The susceptibility of *Escherichia coli* (10^6^ CFU/ml) to honeys or ampicillin (sodium salt) was analyzed by the broth microdilution assay in a 96 well microtitre plate format using serial, two- fold dilution, as previously described [9]. Bacterial growth was monitored at A_595_ nm using the Synergy HT multidetection microplate reader (Synergy HT, Bio-Tek Instruments, Winooski, VT, USA). The MIC was established to be the concentration of honey or ampicillin that reduced bacterial growth by 90% in comparison to a control, untreated culture, after 18 h incubation with shaking at 37°C. Statistical analysis and dose response curves were obtained using K4 software provided by Synergy HT (Bio-Tek Instruments, Winooski, VT, USA).

### General experimental design

*E. coli* culture (10^6^ CFU/ml in 110 µl) were grown in 96-well plates until they reach log phase (A_595_nm 0.2-0.3) at which point honeys or ampicillin was added in triplicate to separate wells at 1xMIC and 0.5x MIC concentrations. At indicated time intervals, the aliquots were taken for incubation wells to examine (a) growth inhibition kinetics (b) total viable counts by the standard plate count, (c) morphological changes and (d) for flow cytometry. The total incubation time was 18 hr at 37°C.

### Time-kill kinetics

After bacterial cultures reached exponential growth, the inhibitory action and killing rate were measured simultaneously at every 20 to 30 min for 2 h and at the end 18 h incubation. The generation time of 20 min for *E. coli* (ATCC 14948) has been established experimentally under conditions used in our laboratory. In a separate experiment, log phase cultures of *E. coli* were first diluted with warm MHB to obtain 10^6^ CFU/ml (using 0.5 McFarland standard), placed in 96-well microplates (100 µl/well) to which honeys or ampicillin were added at 1xMIC or 0.5x MIC. The inhibitory action and killing rate were measured every 20 min using microplate reader. The killing curves were constructed by withdrawing 10 µl aliquots from wells containing inoculum (assay control) and experimental wells. The 10 µl aliquots were serially 10-fold diluted with sterile water to obtain cell density ranging from 10^4^ to 10^2^ CFU/ml and then a 10 µl and 100 µl aliquot from each dilution were plated onto Mueller-Hinton agar (MHA) plates. Since the kinetic results did not differ between 20 min and 30 min of incubation times, we combined the data obtained from these two sets of experiments (6 separate experiments, n = 18).

After 18 hr incubation at 37°C, the viable cells were enumerated. The MBC endpoint was determined as the minimum concentration of glycoproteins at which 99.9% of the initial inoculum was eradicated and at which only one or no colonies could be seen on MHA.

### Cell morphology examination by light microscopy

To examine phenotype changes in *E. coli* induced by honey or ampicillin at different growth phases, 10 µl samples were removed from the experimental and control wells at each time interval at log-phase and at stationary phase (18 hr incubation) from the 96 well plates. The samples were examined on glass slides at 400 x magnifications under light microscope (Zeiss, Axiolab, Germany). Images were viewed and photographed using the digital camera and built-in software (Singer Instruments MSM 400, Somerset, UK).

### Scanning electron microscopy

A log-phase *E. coli* (100 µl, ∼10^8^ CFU/ml) was treated with ampicillin (2.5 µg/ml) or honey H208 (25%w/v) for 3 hr, at 37°C. Cells were harvested by centrifugation (1000×g, 3 min), washed 3 times with filtered-sterilized 0.1 M Tris-buffered saline (TBS, pH 7.3) to remove culture medium, re-suspended in 500 µl of 2.5% glutaraldehyde in 0.1 M TBS (EM grade, Sigma-Aldrich) and incubated for 30 min at room temperature. Cells were washed with 0.1 M TBS and re-suspeded in 100 µl of 0.1 M TBS. Samples (50 ul) were placed on a on a poly-L-lysine coated glass coverslip and left for 60 minutes at room temperature. The samples were dehydrated (2×2 min) in a graded ethanol (70%, 95% and 100%), critical point dried and then sputter coated with gold. The samples were examined using a Hitachi S-530 scanning electron microscope operating at 20 kV. Images were captured using Quartz PCI version 8 software.

### Sample preparation for flow cytometry

The Live/Dead BacLight Bacterial Viability and Counting Kit (L34856, Invitrogen) has been used to evaluate *E. coli* cells viability after their exposure to honey and ampicillin. This kit contains two nucleic acid-specific dyes: Syto9 is membrane-permeable, will stain all cells and can be detected by green fluorescence, and propidium iodide (PI) which is membrane-impermeable, stains cells with damaged membranes and gives red fluorescence. “Log phase” cultures were obtained from time-kill experiments, and were tested after 2 h incubation from the time of honey application to the exponentially growing cells. “Lag phase” cultures resulted from 18 h incubation of lag phase *E. coli* with honey and ampicillin. Both log phase and lag phase *E. coli* cells were treated with honeys H208 and H210 at 1xMIC and 0.5xMIC or ampicillin at concentrations 2.5, 1.25, 0.625 and 0.31 µg/ml. Cultures from the time-kill experiments were collected from 8 replicate wells of 96-well microplates into separate eppendorf tubes, harvested by centrifugation (1,000 *g* for 3 min) washed three times with filtered-sterilized 0.1 M Tris-buffered saline (TBS, pH 7.3) to remove culture medium, resuspended in 300 µl of TBS and double- stained with Syto9 (5 µl final concentration) and PI (30 µl final concentration). Untreated lag phase and log phase cultures were stained only with Syto (live cells) and isopropanol-killed cells were labeled with PI only (dead cells). Samples were incubated at room temperature in the dark for 20 min. After staining, cells were fixed in 100% ethanol overnight at 4°C. Prior flow cytometry, cells were centrifuged and resuspended in TBS.

### Flow cytometric analysis

Untreated *E. coli* cells of intact membranes stained with Syto9 were used as a positive control of live cells, while *E. coli* cells killed with 70% isopropyl alcohol and stained with PI comprised a positive control of dead cells. These cells were used to gate the sorting of the stained cells into four distinct *E. coli* subpopulations: PI- positive (dead cells), Syto-positive (live cells), Syto9/PI double positive cells as injured, dying cells and Syto9/PI double negative cells that may include cell debris. Unstained control *E. coli* culture was also used to compensate for unspecific sorting. The quantitation of cell viability was based on the ratio of green fluorescence of Syto 9 (live cells) to red fluorescence of PI (dead cells) using Live/Dead BacLight Kit (Invitrogen).

The cell populations upon staining with Syto9 and PI were analyzed on a multi-laser flow cytometer BD LSR II (Becton Dickinson, San Jose, CA, USA) by forward and side scatter measurements using fluorescein isothiocyanate filter (green fluorescence) and propidium iodide channels (red fluorescence). BD FACS software was used for data acquisition and analysis. Both fluorescence emission signals were compensated to adjust for overlapping emission signals from the two fluorophores. Microsphere beads (6 um in diameter) were used in the flow cytometry acquision.

### Construction of ampicillin resistant *E. coli*

One hundred µl of an overnight *E. coli* culture was inoculated into 10 ml of Luria Broth (LB: 1% tryptone, 1% NaCl, 0.5% yeast extract) and grown to an OD_600_ of 0.1. The cells were cooled on ice for 10 min, centrifuged (1500 *g*, 5 min, 4°C) and suspended in 10 mL of TBS (Sigma) containing 50 mM CaCl_2_. The suspension was removed in aliquots of 100 µL and pUC19 (New England Biolabs) was added to a final concentration of 4 µg/mL. The cells were incubated on ice for 30 min, heat shocked (42°C, 45 sec) and returned to the ice for 2 min. Nine hundred µL of LB was added to the cells before a 60 min incubation at 37°C with shaking at 250 rpm. The *E.coli* suspension (0.1 mL) was spread on LB plates (2% agar) containing 100 µg/mL ampicillin and grown overnight. Transformants containing the plasmid, identified as growing colonies, were selected and sub-cultured on LB with 100 µg/mL ampicillin.

### Endotoxin release from *E. coli* after treatment with honey and ampicillin

Sterile, pyrogen-free plasticware and water was used for these assays.

The log-phase *E. coli* cultures (∼10^8^ CFU/ml) were incubated with two-fold serially diluted honeys H208 and H210 (25% (w/v) to 6.25% (w/v)) and ampicillin at concentrations 2.5 µg/ml to 0.625 µg/ml (in triplicate) at 37°C for 3 hr in 96-well plates. Cells were harvested by centrifugation (3000×g for 10 min). Samples of cell supernatants were four-times diluted with pyrogen-free water and free endotoxin levels were quantified using a chromogenic Limulus amebocyte lysate (LAL) assay according to the manufacturer instructions (Pierce^R^ LAL chromogenic endotoxin quantitation kit, Pierce Biotechnology, Rockford, IL, USA). The standard curve was constructed according to manufacturer instruction.

### LC-ESI-MS

To determine potential presence of residues of ampicillin in honey, honey samples (5 g) was dissolved in 5 ml sterile, Mili-Q water by vortexing and extracted with 5 ml of ethyl acetate. After centrifugation at 3200 rpm for 5 min, the upper organic layer was collected. The extraction was repeated three times and collected organic layers were dried down with N_2_ flow at 50°C. The extract was reconstituted into 5 ml of sterile, Mili-Q water and purified by solid-phase extraction. Honeys were spiked with amplicillin (stock solution 5 mg/ml) to obtain the final concentration of 250 ng/ml, 500 ng/ml and 1000 ng/ml. The presence of ampicillin in unspiked honeys and spiked honeys were quantified by liquid chromatography coupled to electrospray ionization mass spectrometry (LC-ESI-MS) operating in negative mode (Table 2). Ampicillin was used as a standard. The method was validated by achieving reproducible, quantitative results.

**Table 2:** Condition for ampicillin quantification in unspiked and spiked honeys using LC-ESI-MS.

Time (min)	Solvent A (%)	Solvent B (%)
0.00	95	5
2.00	95	5
12.00	20	80
15.00	10	90
20.00	10	90

Column: Eclipse XDB-C18/4.6×75 mm/3.5u.

Flow: 0.250 ml/min.

Solvent A: water with 0.1% formic acid.

Solvent B: acetonitrile with 0.1% formic acid.

### Statistical analysis

All data were presented as mean values ± SD. Groups were compared with unpaired t-test using Graph Pad InStat version 3.00, GraphPad Software Inc., San Diego California USA. The data were tested for normality of distribution and they passed the test. Differences between means were considered to be significant at p<0.05.
